# Association between the Angle of the Left Subclavian Artery and Procedural Time for Percutaneous Coronary Intervention in Patients with Acute Coronary Syndrome

**DOI:** 10.1155/2022/3249745

**Published:** 2022-11-17

**Authors:** Masatsugu Miyagawa, Daisuke Fukamachi, Katsunori Fukumoto, Masaki Monden, Kurara Takahashi, Shohei Migita, Saki Mizobuchi, Yudai Tanaka, Akihito Ogaku, Yutaka Koyama, Hidesato Fujito, Riku Arai, Norio Takei, Keisuke Kojima, Korehito Iida, Nobuhiro Murata, Yasuo Okumura

**Affiliations:** Division of Cardiology, Department of Medicine, Nihon University School of Medicine, Tokyo, Japan

## Abstract

**Background:**

The effect of left subclavian artery tortuosity during percutaneous coronary intervention (PCI) in patients with acute coronary syndrome (ACS) remains unclear.

**Methods:**

Of 245 ACS patients (from November 2019 and May 2021), 79 who underwent PCI via a left radial approach (LRA) were included. We measured the angle of the left subclavian artery in the coronal view on CT imaging as an indicator of the tortuosity and investigated the association between that angle and the clinical variables and procedural time.

**Results:**

Patients with a left subclavian artery angle of a median of <70 degrees (severe tortuosity) were older (75.4 ± 11.7 vs. 62.9 ± 12.3 years, *P* < 0.001) and had a higher prevalence of female sex (42.1% vs. 14.6%, *P*=0.007), hypertension (94.7% vs. 75.6%, *P*=0.02), and subclavian artery calcification (73.7% vs. 34.2%, *P* < 0.001) than those with that ≥70 degrees. The left subclavian artery angle correlated negatively with the sheath cannulation to the first balloon time (*ρ* = −0.51, *P* < 0.001) and total procedural time (*ρ* = −0.32, *P*=0.004). A multiple linear regression analysis revealed that the natural log transformation of the sheath insertion to first balloon time was associated with a subclavian artery angle of <70 degrees (*β* = 0.45, *P* < 0.001).

**Conclusion:**

Our study showed that lower left subclavian artery angles as a marker of the tortuosity via the LRA were strongly associated with a longer sheath insertion to balloon time and subsequent entire procedure time during the PCI.

## 1. Introduction

Radial access percutaneous coronary intervention (PCI) is associated with a reduction in clinical adverse events in comparison to a femoral access in patients with acute coronary syndrome (ACS) [1–5]. The right radial approach (RRA) is favored by many operators because of the ease of a standard access setup [6]. However, the RRA is also associated with technical difficulties due to the right subclavian artery tortuosity in comparison to the left radial approach (LRA) [7]. Furthermore, arterial anatomic variations including the tortuosity of the right subclavian artery influence the transradial procedural duration and outcomes [8, 9]. Recent studies have shown that the use of a LRA in the management of ST-elevated myocardial infarction (STEMI) patients is associated with a comparable success rate and reperfusion times when compared with the RRA [6]. Therefore, the LRA can be an alternative approach to the RRA in some ACS patients, and it may be preferable for right-handed persons when considering the vascular complication risk. Although it is physically clear that the tortuosity of the left subclavian artery affects the passing of the wire and catheter or engaging the catheter, the data on the impact of the tortuosity of the left subclavian artery on the time required for the PCI in patients with ACS are lacking. Because it is well known that treatment delays are important determinants of patient outcome in patients with ST-elevation acute myocardial infarction (STEMI) [10] and some patients with non-STEMI (NSTEMI) [11], the identification of challenging cases through an LRA would be important. We hypothesized that the angular change in the left subclavian artery detected on chest computed tomography (CT) due to tortuosity influences the time of the PCI using the LRA in patients with ACS. We, therefore, aimed to characterize the patients with severe tortuosity of the left subclavian artery and to investigate the association between the angular changes in the left subclavian artery and the time for the PCI.

## 2. Methods

### 2.1. Study Patients

**Figure 1:**
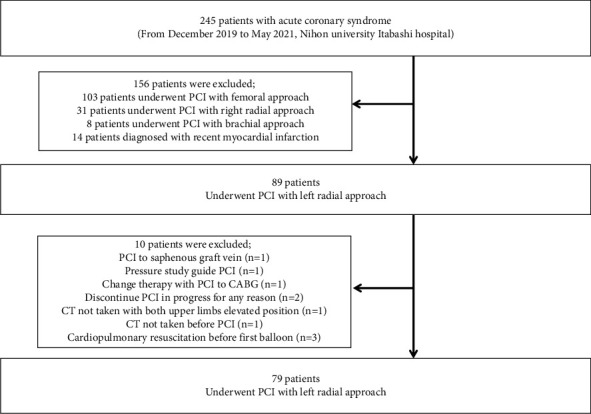
Study flow diagram. CABG, coronary artery bypass grafting; PCI, percutaneous coronary intervention.

This was a retrospective observational study of 245 consecutive patients who underwent PCI for ACS with single-plane imaging at Nihon University Itabashi Hospital, Tokyo, Japan, between December 2019 and May 2021. The study flow diagram is shown in Figure 1. Patients who had undergone a PCI via an LRA after a CT scan on admission were included in this study. In our hospital, an LRA is the first-choice approach for coronary angiography or PCI unless there is a reason such as a left-handed person, an arterial line being inserted, a paralyzed side, or a poor radial pulse on palpation. The LRA included only a conventional and not a distal LRA. We did not consider CT to be necessary in all patients with ACS. However, this study was performed during the COVID-19 pandemic period. So, in our hospital, patients admitted to the hospital were subjected to a CT scan on admission as much as possible for the detection of mild or asymptomatic infections [12]. Patients who met any of the following criteria were excluded: (1) patients who had undergone a PCI via other arterial access approaches in such patients as those with dialysis; (2) recent myocardial infarction patients whose onset-time was from 2 to 28 days [13]; (3) patients who underwent a PCI to a non-native coronary vessel; (4) patients undergoing a physiological testing (instantaneous wave-free ratio/fractional flow reserve) guided PCI; (5) those in whom the PCI was terminated for any reason; and (6) other reasons, as shown in Figure 1. In this study, no patients had a history of left subclavian artery trauma. According to those criteria, of the 245 ACS patients, a total of 79 were included in this study. The study protocol was approved by the Ethics Committee of Nihon University Itabashi Hospital (RK-201121-01) and was in accordance with the ethical standards of the institutional research committee and the 1964 Declaration of Helsinki and its later amendments or comparable ethical standards.

### 2.2. Measurement of the Angle of the Left Subclavian Artery and the Aortic Root

**Figure 2:**
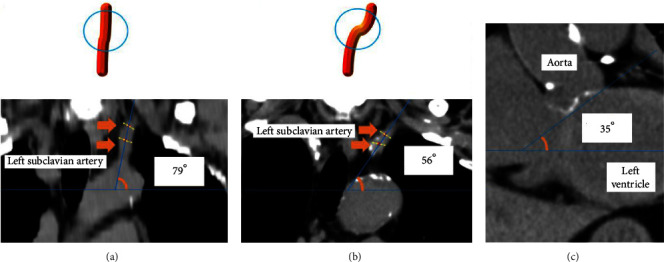
Representative schematic images (upper panels) and CT images in the coronal view (lower panels) in patients with mild tortuosity (a) and severe tortuosity (b). (a) In a case of mild tortuosity, the angle of the left subclavian artery is 79 degrees. In this case, the time from sheath insertion to the first balloon time was 20 minutes and total procedure time was 46 minutes, respectively. (b) In patients with severe tortuosity, the angle of the left subclavian artery was 55 degrees. In this case, the time from sheath insertion to the first balloon time, the total procedure time, and the fluoroscopy time were longer than in case (a) (43 minutes and 97 minutes, respectively). (c) The angle of the aortic root on the coronal view. The details of the measurement method are shown in the text. LRA, left radial approach; PCI, percutaneous coronary intervention.

Multislice helical CT (Canon Medical Systems, Tokyo, Japan) was performed with both upper limbs elevated at the time of admission or after the PCI. A prior study on vascular models with/without tortuosity reported by Weiss et al. identified that the angles differ as a result of the tortuosity [14], and therefore, the angle of the left subclavian artery in the thorax was measured as an indicator of the tortuosity with the following method (Figures 2(a) and 2(b)). The coronal plane image from the point where the bifurcation of the vertebral artery as a geographic landmark in the thorax was used for the measurement of the left subclavian artery. First, the coronal plane image that clearly identified the left subclavian artery was chosen. Secondly, two points at least 5 mm away from each other were selected in the center of the blood vessel in the left subclavian artery. Thirdly, the angle of the line connecting the two points to the horizontal plane line was measured. Finally, the above measurements were repeated twice, and the lower angle was selected. The angle of the left subclavian artery was estimated by two independent expert cardiologists who were not provided with the patients' clinical information. Two independent observers measured the angles of the left subclavian artery in the coronal CT in 25 randomly selected subjects to assess the interobserver reproducibility (intraclass correlation coefficients, 0.71), indicating a good reproducibility. A previous study indicated an association between the aortic root angle and success rates of transcatheter aortic valve implantations [15]. The angle of the aortic root was also measured on the coronal plane CT as previously reported (Figure 2(c)) [16].

### 2.3. Severity Assessment of the Coronary Artery Disease

The ACS included STEMIs, NSTEMIs, and unstable angina (UA) according to the 4th universal definition of myocardial infarction [17]. Stenosis was considered significant if it was 75% according to the American Heart Association guidelines. A high-lateral culprit lesion was classified as a left circumflex artery and a diagonal branch as a left anterior descending artery. The definition of the thrombolysis in myocardial infarction trial (TIMI) flow was graded as TIMI 0 = no perfusion, TIMI 1 = penetration without perfusion, TIMI 2 = partial perfusion, and TIMI 3 = complete perfusion, as described in the Phase I TIMI Trial [18].

### 2.4. Radial Artery Cannulation

Radial artery punctures were performed with a dedicated radial cannulation needle and guidewire after local subcutaneous anesthesia with 1% lidocaine. A 6 French short hydrophilic sheath (Radifocus® Introducer II, Terumo, Japan) was inserted, and 5000 IU of heparin was given.

### 2.5. Transradial Coronary Angiography and Percutaneous Coronary Intervention

The examination and treatment plan were based on the current guidelines [19]. For coronary angiography, the catheters to be employed were a 5 French Judkins right 4.0 (Goodtech, Nipro, Japan) for access to the right coronary artery and a 5 French Judkins left 4.0 (Goodtech, Nipro, Japan) for access to the left coronary artery. In the case of the inability to engage the coronary ostium, different sizes of Judkins or Amplatz catheters were used according to the operator's preference. The procedure started with a 0.035-inch standard guidewire (the Swan Excel Guide Wire, ALT, Japan). Other wires were used as the operator required. For the PCI, a 6 French guiding catheter that the operator preferred was employed for the access to the culprit coronary artery after coronary angiography. Backup catheters included the following: Super power backup (SPB, ASAHI INTECC, Japan), CLS™ Curve (CLS, Boston Scientific, USA), Short Amplatz Left (SAL, ASAHI INTECC, Japan), and Amplatz Left (AL, ASAHI INTECC, Japan). These catheters were all side-hole types. The primary PCI was performed with intravascular ultrasound (IVUS) (OPTICROSS, Boston Scientific, USA) guidance and in accordance with the standard technique and current guidelines for single-plane machines. Side branch protection was defined as when a wire was placed in a branch other than the main trunk at the stent placement site or plain old angioplasty. The stent included second- and third-generationdrug-eluting stents (Xience family, Abbott Vascular, Santa Clara, California; Synergy, Boston Scientific, Natick, Massachusetts; Resolute Onyx, Medtronic, Santa Rosa, CA; Ultimaster™ Tansei™Terumo, Tokyo, Japan; or Orsiro stent, Biotronik, Bülach, Switzerland). The contrast medium used was iomeprol (Iomeron, Bracco Eizai, Japan) unless there was a reason not to use it. Nonhighly experienced operators were defined as a personal experience of <100 PCI cases. All patients received aspirin (200 mg) and a loading dose of clopidogrel (300 mg) or prasugrel (20 mg) if they were not receiving dual antiplatelet therapy.

### 2.6. Endpoints and Assessments

The endpoints were the total procedure time, sheath cannulation to the first balloon time, and incidence of contrast-induced nephropathy (CIN) as previously reported [6, 20, 21]. The procedure time was defined as the interval from the sheath cannulation into the left radial artery to the end of the PCI. Sheath cannulation to balloon time was defined as the interval from the sheath cannulation to the first balloon dilation or first thrombus aspiration. CIN was defined as an increase in the serum creatinine of ≥0.3 mg/dl or ≥50% within 72 hours from the PCI [22].

### 2.7. Statistical Analysis

The categorical variables were presented as numbers and percentages and compared between the groups using a chi-square test or Fisher exact test as appropriate. The continuous variables were expressed as the mean ± SD or median with the 25th and 75th percentiles and compared using a Student's *t*-test or Wilcoxon rank sum test, as appropriate. Because the total procedure time and sheath cannulation to the first balloon time were not normally distributed, a Spearman regression analysis was performed to find the association between those times and the angle of the subclavian artery and between the sheath cannulation to the first balloon time and clinical variables. A multivariate regression analysis was performed to find the significant variables by a single regression analysis and natural log transformation of the sheath insertion to the first balloon time. A *P* value of 0.05 was considered statistically significant. Those analyses were performed with JMP Pro 16 software (SAS Institute, Cary, NC, USA).

## 3. Results

### 3.1. Patient Characteristics

The patient characteristics are listed in Table 1. The mean age was 68.9 ± 13.5, and 27.8% of the patients were female. The frequencies of a history of hypertension and PCI were 84.8% and 15.2%, respectively. None of the patients had a history of a coronary artery bypass graft. In this study, STEMIs were the most frequent type of ACS (65.8%) and the left descending artery (LAD) was often the culprit lesion (43.6%). Not many patients had three-coronary vessel disease (35.4%). The median time from sheath cannulation to the first balloon time and total procedure time were 27.6 ± 13.1 min and 69.9 ± 39.6 min, respectively. All patients except for two had 3rd generation drug-eluting stents implanted.

**Figure 3:**
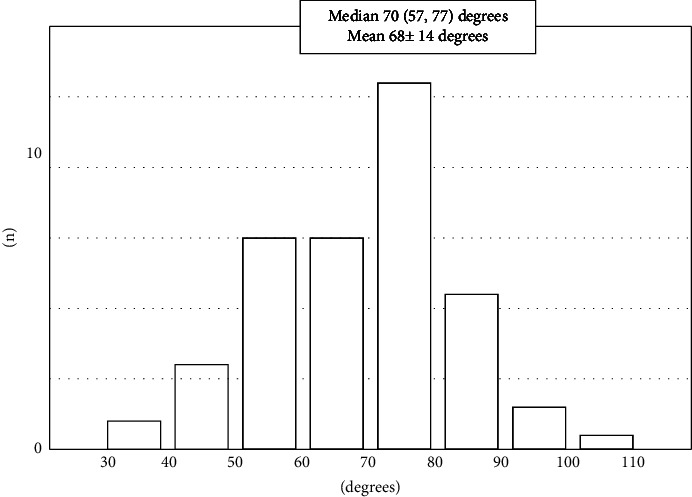
Distribution of the angle of the subclavian artery in the coronal view. The median value was 70 (57, 77) degrees, and the mean value was 68 ± 14 degree.

The distribution of the angle of the subclavian artery in the coronal view was normally distributed, and the median value was 70 degrees (57, 77), and the mean was 68 ± 14 degrees (Figure 3). According to the median value, the patients were divided into two groups including those with <70 degrees (*n* = 38) and those ≥70 degrees (*n* = 41), respectively (Table 2). The <70 degree patients had a higher age (75.4 ± 11.7 vs. 62.9 ± 12.3 years, *P* < 0.001) and a higher prevalence of being female (16 [42.1%] vs. 6 [14.6%], *P*=0.007), history of hypertension (36 [94.7%] vs. 31 [75.6%], *P*=0.02), and presence of subclavian artery calcifications (28 [73.7%] vs 14 [34.2%], *P* < 0.001) than the ≥70-degree patients. There was no difference in the angle of the aortic root between the two patient groups (45.5 ± 9.2 vs. 42.9 ± 7.9 degrees, *P*=0.17). Regarding the PCI-related findings, patients with <70 degrees exhibited a longer procedure time (81.3 ± 45.2 vs. 59.4 ± 30.5 min, *P*=0.013) and sheath insertion to the first balloon time (34.4 ± 14.1 vs. 21.4 ± 8.3 min, *P* < 0.001) than the ≥70 degree patients. In patients with culprit lesions of the right coronary artery (RCA) (*n* = 25), the patients with an angle of <70 degrees exhibited a longer sheath insertion to the first balloon time (20.9 ± 8.9 vs. 12.7 ± 2.0 min, *P*=0.009) and procedure time (55.5 ± 35.1 vs. 26.4 ± 7.1 min, *P*=0.017) than the ≥70 degree patients. In patients with a culprit left coronary artery (LCA) (*n* = 54), the patients with an angle of <70 degrees also exhibited a longer sheath insertion to the first balloon time (20.5 ± 8.5 vs. 12.9 ± 5.7 min, *P* < 0.001), but the procedural time did not significantly differ between the two patient groups (44.4 ± 20.1 vs. 38.6 ± 19.8 min, *P*=0.30).

### 3.2. Associations between the Angle of the Subclavian Artery on the Coronal View and PCI-Related Parameters

**Figure 4:**
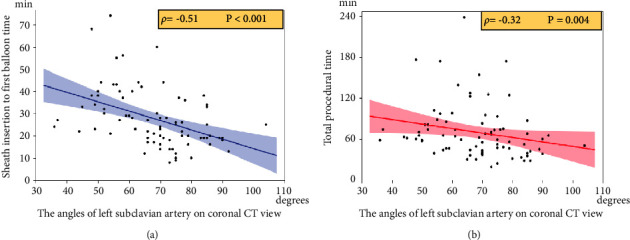
Correlations between the angle of the left subclavian artery in the coronal view and sheath insertion to the first balloon time (a) and total procedural time. (b) The details are shown in the text.

**Figure 5:**
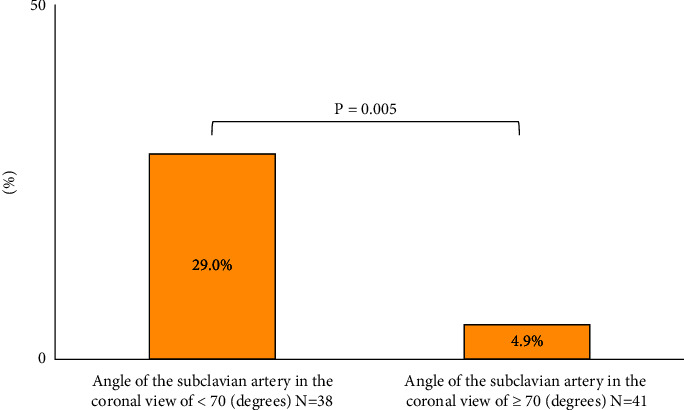
Incidence of contrast-induced nephropathy. Contrast-induced nephropathy occurred more frequently with an angle of the subclavian artery in the coronal view of <70 degrees than of ≥70 degrees (11 patients; 29.0% vs. 2 patients; 4.9%, *P*=0.005).

The angle of the subclavian artery in the coronal view correlated negatively with the sheath insertion to the first balloon time (*ρ* = −0.51, *P* < 0.001) and total procedural time (*ρ* = −0.32, *P*=0.004) (Figures 4(a) and 4(b)). A creatinine assessment after the PCI was performed in all patients at 24 hours, 92% of the patients at 48 hours, and 95% of the patients at 72 hours. CIN occurred more frequently for an angle of the subclavian artery in the coronal view of <70 degrees (11 patients, 29.0%) than for ≥70 degrees (2 patients, 4.9%) (Odds ratio 7.78, 95% CI 1.64–38.74, *P*=0.005) (Figure 5).

### 3.3. Correlations between the Sheath Insertion to the First Balloon Time and Clinical Variables

The time from sheath insertion to the first balloon time was a median of 25 (19, 36: ranges 8–74) minutes. The Spearman rank correlations of the clinical factors with the sheath insertion to the first balloon time are shown in Table 2. Three-coronary vessel disease and a nonhighly experienced operator correlated with the sheath insertion to the first balloon time. A pre-TIMI flow grade of 3 was more likely to be correlated with the sheath insertion and the first balloon time. A multiple regression analysis revealed that the natural log transformation of the sheath insertion to the first balloon time remained to be significantly associated with a lower angle of the subclavian artery on the coronal view (*β* = −0.38, *P* < 0.001) or an angle of the subclavian artery of <70 degrees (*β* = 0.45, *P* < 0.001) even after adjusting for the three-coronary vessel disease, nonhighly experienced operator, and a pre-TIMI flow grade of 3.

## 4. Discussion

This study had two major findings: (1) a lower angle of the left subclavian artery was related to an older age, female sex, and the presence of hypertension in patients with ACS who underwent a left radial PCI; (2) a lower angle of the left subclavian artery have a moderate correlation with a longer sheath insertion to the first balloon time and an incidence of CIN after the PCI, and it was independently associated with a longer sheath insertion to the first balloon time even after the multivariate adjustment.

### 4.1. The Factors and Clinical Importance Associated with the Angle of the Subclavian Artery in Patients with ACS

Although it is well-known that a tortuous artery is associated with difficulty and complications during catheterization procedures [23], measuring and reporting the tortuosity of an artery remains a challenging task in the absence of a standardized, universally accepted method [24]. In the most recent study, the right subclavian artery tortuosity has been defined as experimental manipulation difficulty [25]. Measurements at the points of angulation of the intracerebral vasculature from magnetic resonance angiography images can partly evaluate the arterial tortuosity [26], and an experimental computational model has indicated that the angles differ as a result of the tortuosity [14]. Because the tortuosity of an artery is difficult to assess with angiography, those prior reports [14, 26] have shown that our new method that quantitatively assesses the left subclavian artery angle on CT imaging is an indicator of the artery tortuosity. We do not believe that CT scans prior to cardiac catheterization are necessary for all patients with ACS. However, the angle assessment on CT imaging may provide a clinical advantage over the other modalities, especially in patients with ACS because ACS patients often undergo a CT prior to the PCI to detect and evaluate any aortic dissections or aortic aneurysms [27, 28]. In addition, a CT scan taken previously would be informative, even if not immediately prior to the cardiac catheterization. The RRA is widely used because of the associated ease and familiarity of the equipment manipulation, but the data regarding the LRA are lacking. We newly found that the left subclavian artery tortuosity as represented by a lower angle of the left subclavian artery was significantly associated with an older age, female sex, and the presence of hypertension. Cha et al. retrospectively investigated the factors associated with severe tortuosity in 2,341 consecutive patients who underwent an initial coronary angiography via the right radial artery. Their study also showed that the clinical predictors of severe tortuosity of the right subclavian artery were hypertension, a female gender, an older age, and a short stature [25]. Therefore, although it has been expected, the clinical predictors of severe tortuosity may be similar regardless of a right or left subclavian artery.

There were only a few studies that have investigated the time from sheath insertion to the first balloon time, fluoroscopy time, and total procedural time in a primary PCI for ACS, especially the LRA, because many studies have investigated the time from door to balloon time as an important factor in the treatment of STEMIs. Fu et al. investigated the efficacy of the LRA as compared to the RRA for a primary PCI in STEMI patients [20]. Their sheath cannulation-to-balloon time via the LRA was 16.0 ± 4.8 minutes and contrast medium volume was 125.8 ± 19.6 mL, respectively. Although their contrast medium volume was similar to our results (136.7 mL), our sheath cannulation-to-balloon time was longer (27.6 ± 13.1). The plausible reason for the slightly longer time in our study was that we confirmed the morphology of the lesion with IVUS before the first balloon dilation as much as possible. Nonetheless, their study did not investigate the association between the clinical factors and sheath insertion to the first balloon time via the LRA [20]. In patients with culprit lesions of the LCA, the procedural time did not significantly differ between an angle of the subclavian artery of <70 degrees and ≥70 degrees. The plausible reason for the minimal effect of the angle on the procedural time was that the LCA included the LAD, which is longer than the RCA or circumflex artery and requires many stents. In our study, multiple stent implantations were more common in the patients with culprit lesions of the LCA than of the RCA (20 [37.0%] vs. 7 [28.0%], *P*=0.43). Moreover, in the group with an LCA culprit, the prevalence of multiple stent implantations was less in the <70 degree than ≥70 degrees group (7 [30.4%] vs. 13 [41.9%], *P*=0.38).

In this study, a univariate analysis showed that three-coronary vessel disease, not highly experienced operators, and a pre-TIMI flow grade 3 were more likely to be associated with a long sheath insertion to the first balloon time; however, it was not evident for the potential factors such as diabetes, the lesion length, severe calcifications, etc., which were derived from the data to explore the associated factors of the long fluoroscopy time [29] via mixed approaches including the radial artery, femoral artery, or rarely, the brachial artery. Although the exact explanation of those 3 associated factors was unclear, three-coronary vessel disease and a pre-TIMI flow grade 3 may be confounders of severe coronary disease and/or severe tortuosity, and a pre-TIMI flow grade 3 may be an inverse consequence of physicians having hurriedly performed the revascularization in patients who had a pre-TIMI follow grade of ≤2. More importantly, this study revealed that a lower angle of the left subclavian artery correlated significantly with a longer sheath insertion to the first balloon time and procedure time, and it remained an independent factor of a longer sheath insertion to the first balloon time. This implicated that the arterial tortuosity was strongly associated with the difficulty not only in the manipulation while passing the wire and catheter or engaging the catheter but also to treat the coronary arteries themselves. This was suggested by the higher requirement of microcatheters for the culprit lesion wire crossing and the presence of subclavian artery calcifications in patients with an angle of the left subclavian artery of <70 degrees.

Our study also showed that patients with an angle of the left subclavian artery of <70 degrees had a risk of an incidence of CIN. Several risk factors for CIN are associated with atherosclerosis [30]. Therefore, the angular changes in the left subclavian artery are considered to be the representation of those risk factors on the CT image.

### 4.2. Clinical Implications

When the angle of the left subclavian artery is lower, it is not necessary to change the LRA to another approach. According to previous studies, the clinical predictors of severe tortuosity may be similar regardless of whether a right or left subclavian artery or brachiocephalic artery [25]. Therefore, physicians should more carefully manipulate the catheters and exchange the guidewires or catheters, and they should not apply undue force in such patients in order to prevent severe vascular complications. A lower angle of the subclavian artery with the risk of a longer procedure time is valuable in patients with ACS who are sometimes hemodynamically unstable because it is helpful for deciding whether to administer mechanical support and invasive ventilation before the revascularization. In the patients with an angle of the left subclavian artery of <70 degrees, the incidence of CIN was significantly higher than in the ≥70 degrees group, so contrast medium should be minimized to prevent contrast nephropathy.

## 5. Limitations

There were several limitations to our study. First, the present study included a small size and single-center sample. Second, this was a retrospective study of patients who were selected for the LRA. Therefore, the analysis did not include dialysis patients or a group of patients whose radial artery was not touched, who were considered to have a higher degree of atherosclerosis than the subjects in this study. Third, the CT images were taken when the patient's hands were positioned above the head, so it should be considered that our angle measurements were obtained under this patient's position. Finally, patients who did not undergo CT scans before the PCI were not applicable. Nonetheless, we found significant associations between a lower angle of the left subclavian artery and being female, a low height, and hypertension, and thus, such factors may help identify patients who have the potential risk for a long sheath insertion before the first balloon time.

## 6. Conclusion

Our study showed that lower angles of the left subclavian artery as a marker of the tortuosity via an LRA were strongly associated with a longer sheath insertion time to the first balloon time and subsequent entire procedure time during the PCI.

## Figures and Tables

**Table 1:** Patient characteristics per study group.

Variable	Total *N* = 79	Angle of the subclavian artery in the coronal view of <70 (degrees) *N* = 38	Angle of the subclavian artery in the coronal view of ≥70 (degrees) *N* = 41	*P* values
Age (years)	68.9 ± 13.5	75.4 ± 11.7	62.9 ± 12.3	<0.001
Female sex	22 (27.8%)	16 (42.1%)	6 (14.6%)	0.007
Height (cm)	162.3 ± 9.5	159 ± 9.5	164.7 ± 8.8	0.03
Body mass index (kg/m^2^)	23.4 ± 3.2	23.4 ± 3.9	24.6 ± 3.6	0.17
Comorbidity				
Hypertension	67 (84.8%)	36 (94.7%)	31 (75.6%)	0.02
Diabetes	28 (35.4%)	14 (36.8%)	14 (34.1%)	0.80
Dyslipidemia	51 (63.8%)	21 (55.3%)	29 (70.7%)	0.15
Smoking history	49 (62.0%)	23 (60.5%)	26 (63.4%)	0.79
Previous PCI	12 (15.2%)	7 (18.4%)	5 (12.2%)	0.54
Statin	24 (30.3%)	10 (26.3%)	14 (34.1%)	0.45
Laboratory examination				
HbA1c (%)	6.3 ± 1.1	6.3 ± 0.9	6.2 ± 1.3	0.73
T-chol (mg/dL)	207.8 ± 53.6	201.0 ± 48.5	213.9 ± 57.8	0.30
LDL (mg/dL)	124.9 ± 47.2	116.8 ± 43.9	132.0 ± 50.3	0.17
HDL (mg/dL)	47.9 ± 12.3	46.8 ± 12.5	49.0 ± 12.4	0.42
TG (mg/dL)	122 (87, 169)	118 (82, 195)	122 (89, 166)	0.88
Creatinine	0.94 ± 0.41	0.95 ± 0.47	0.94 ± 0.35	0.97
Estimated glomerular filtration rate at baseline (mL/min/1.73 m^2^)	58.9 ± 25.2	64.1 ± 32.5	54.0 ± 14.6	0.07
Peak creatinine kinase in patients with STEMIs (IU/L)	1245 (434, 2789)	855 (397, 1644)	1943 (472, 3042)	0.06
*N*-terminal prohormone of brain natriuretic peptide (pg/mL)	635 (163, 2400)	890 (253, 3231)	478 (136, 1646)	0.23
Classification of acute coronary syndrome				
STEMI	52 (65.8%)	24 (63.2%)	28 (68.3%)	0.63
Culprit lesion in the LAD	34 (43.6%)	19 (50.0%)	15 (37.5%)	0.27
Three-coronary vessel disease	28 (35.4%)	17 (44.7%)	11 (26.8%)	0.10
Pre-TIMI flow 3	29 (36.7%)	17 (44.7%)	12 (29.3%)	0.15
PCI procedure				
Fluoroscopy time (min)	19.0 (13.6, 32.7)	23.9 (14.5, 38.1)	16.6 (13.3, 28.7)	0.09
Total procedure time (min)	69.9 ± 39.6	81.3 ± 45.2	59.4 ± 30.5	0.013
Sheath insertion to the first balloon time (min)	27.6 ± 13.1	34.4 ± 14.1	21.4 ± 8.3	<0.001
Contrast medium volume	136.7 ± 42.8	140.5 ± 40.2	133.3 ± 47.2	0.46
Changing guiding catheter	7 (8.9%)	5 (13.2%)	2 (4.9%)	0.25
Back up catheter	55 (69.6%)	28 (73.7%)	27 (65.9%)	0.45
Microcatheter used for culprit lesion wire crossing	19 (24.1%)	13 (34.2%)	6 (14.6%)	0.06
Predilation	67 (84.8%)	31 (81.6%)	36 (87.8%)	0.53
Postdilation	57 (72.2%)	25 (65.8%)	32 (78.0%)	0.22
Multiple stent implantation	24 (31.6%)	12 (32.4%)	12 (30.8%)	0.88
Side branch protection	30 (38.0%)	13 (34.2%)	17 (41.5%)	0.51
Aspiration	17 (21.5%)	9 (23.7%)	8 (19.5%)	0.65
Temporary pace maker	21 (26.6%)	11 (30.0%)	10 (24.4%)	0.67
Intra-aortic balloon pumping	10 (12.5%)	5 (13.2%)	5 (12.2%)	1.00
Impella	2 (2.5%)	0	2 (4.9%)	0.49
Not highly experienced operator	13 (16.5%)	8 (21.1%)	5 (12.2%)	0.37
PCI to multiple coronary vessels	9 (11.4%)	4 (10.5%)	5 (12.2%)	1.00
CT finding				
Presence of subclavian artery calcification	42 (53.1%)	28 (73.7%)	14 (34.2%)	<0.001
Angle of the aortic root (degrees)	44.1 ± 8.6	45.5 ± 9.2	42.9 ± 7.9	0.17

Values are the median (25th, 75th quartile). HDL, high density lipoprotein; LAD, left descending artery; LDL, low density lipoprotein; LMT, left main trunk; PCI, percutaneous coronary intervention; STEMI, ST-segment elevation myocardial infarction; T-cho, total cholesterol; TIMI, thrombolysis in myocardial infarction trial; TG, triglyceride.

**Table 2:** Spearman regression analysis of the sheath insertion to the first balloon time.

	Sheath insertion to the first balloon time (min)	
	*ρ*	*P* values
Variables		
Age (years)	0.13	0.27
Female sex	0.07	0.53
Body mass index (kg/m^2^)	0.16	0.15
Comorbidity		
Hypertension	0.16	0.17
Diabetes	0.09	0.42
Dyslipidemia	−0.07	0.54
Smoking history	0.08	0.50
Previous PCI	0.01	0.94
Laboratory examination		
HbA1c (%)	0.04	0.71
T-chol (mg/dL)	−0.21	0.06
LDL (mg/dL)	−0.19	0.09
HDL (mg/dL)	−0.27	0.02
TG (mg/dL)	−0.05	0.66
Creatinine (mg/dL)	−0.13	0.23
Classification of acute coronary syndrome		
STEMI	−0.12	0.26
Culprit lesion in the LAD	−0.04	0.73
Three-coronary vessel disease	0.29	0.001
Pre-TIMI flow grade 3	0.21	0.07
PCI procedure		
Temporary pace maker	0.06	0.61
Intra-aortic balloon pumping	0.02	0.84
Nonhighly experienced operator	0.25	0.02
CT finding		
Angle of the aortic root (degrees)	0.11	0.33

HDL, high density lipoprotein; LAD, left descending artery; LDL, low density lipoprotein; LMT, left main trunk; PCI, percutaneous coronary intervention; STEMI, ST-segment elevation myocardial infarction; T-cho, total cholesterol; TIMI, thrombolysis in myocardial infarction trial; TG, triglyceride.

## Data Availability

The data used to support the findings of this study are available from the corresponding author upon request.
